# Evaluation of predicted Medfly ( *Ceratitis capitata*) quarantine length in the United States utilizing degree-day and agent-based models

**DOI:** 10.12688/f1000research.12817.2

**Published:** 2018-03-06

**Authors:** Travis Collier, Nicholas Manoukis

**Affiliations:** 1 Daniel K. Inouye US Pacific Basin Agricultural Research Center (PBARC), United States Department of Agriculture, Agricultural Research Service, Hilo, Hawaii, USA

**Keywords:** biosecurity, Mediterranean fruit fly, eradication, invasive pest, agriculture

## Abstract

Invasions by pest insects pose a significant threat to agriculture worldwide. In the case of *Ceratitis capitata* incursions on the US mainland, where it is not officially established, repeated detections are followed by quarantines and treatments to eliminate the invading population. However, it is difficult to accurately set quarantine duration because non-detection may not mean the pest is eliminated. Most programs extend quarantine lengths past the last fly detection by calculating the amount of time required for 3 generations to elapse under a thermal unit accumulation development model (“degree day”). A newer approach is to use an Agent-Based Simulation (ABS) to explicitly simulate population demographics and elimination. Here, predicted quarantine lengths for 11 sites in the continental United States are evaluated using both approaches. Results indicate a strong seasonality in quarantine length, with longer predictions in the second half of the year compared with the first; this pattern is more extreme in degree day predictions compared with ABS. Geographically, quarantine lengths increased with latitude, though this was less pronounced under the ABS. Variation in quarantine lengths for particular times and places was dramatically larger for degree day than ABS, generally spiking in the middle of the year for degree day and peaking in second half of the year for ABS. Analysis of 34 *C. capitata* quarantines from 1975 to 2017 in California shows that, for all but two, quarantines were started in the second half of the year, when degree day quarantine lengths are longest and have the highest uncertainty. For a set of hypothetical outbreaks based on these historical quarantines, the ABS produced significantly shorter quarantines than degree day calculations. Overall, ABS quarantine lengths were more consistent than degree day predictions, avoided unrealistically long values, and captured effects of rare events such as cold snaps.

## Introduction

Invasions by insects, pathogens and pests are increasingly a defining challenge of the 21 ^st^ century, facilitated by global connectivity, climatic shifts, and other factors ^1, 2^, with a particularly severe impact on agriculture ^3^. Invasions by insects that do not become established have a lower public profile than those that are “successful” from the point of view of the insect. However, there is a greater chance that cases of invasion followed by elimination will be detected and studied when the invading species is of environmental, human health, or economic concern ^4^. Eradicating local populations of such insects can be desirable and feasible ^5, 6^ depending on several factors.

One factor determining the feasibility of elimination is if the new environment is only marginally or seasonally suitable to the invading insect, facilitating its eradication. Another is when the high cost of allowing establishment leads to extensive efforts for eradication. The invasion of the malaria mosquito species *Anopheles gambiae* into Northeastern Brazil in the 1930’s ^7^ is one example of an invasive insect that was successfully eradicated primarily due to the second of these factors ^8, 9^.

In the case of *An. gambiae* there have been no reports of reinvasion, but there are examples of insects that recurrently invade areas outside their native range and are recurrently eliminated within relatively few generations. The Gypsy moth *Lymantria dispar* in Canada ^10^ is one such species. Arguably, another example is the screwworm *Cochlyomyia hominivorax* along the current northernmost edge of its range in Panama ^11^ and more recently in Florida ^12^.

One of the most important instances of repeated invasion and elimination by an economically important insect pest is that of the Mediterranean fruit fly *Ceratitis capitata* (Wiedemann) (Medfly) in California. The last four decades have seen a repeated pattern of invasion, detection, and response interspersed by periods of no detections ^13, 14^. While it has been suggested that this pattern is the result of cryptic establishment ^15^, the majority view is that Medfly in California is an example of a “metain-vasion”, consisting of multiple sequential or overlapping introductions ^16^ and repeated eradication ^17^. Still other researchers point to the possibility of different situations in different regions of the state ^18, 19^. Medfly is occasionally found in other parts of the mainland US such as Florida ^20^, and in other countries or areas that are considered free of the pest including Eastern Australia, Mexico and Chile ^21^.

The response plan to Medfly in California and the other “free” regions mentioned above is extensive and costly, including a quarantine when detections exceed an established standard (more than a male or unmated female fly is detected) ^22^. Perfect pest surveillance efforts could determine exactly when eradication has been achieved. However, actual surveillance has a density threshold below which it is increasingly probable that a population is undetected. A practical and important problem is how long to maintain the countermeasures and quarantine after flies are no longer detected. Predicting the likely duration of this ‘post last detection’ quarantine period (hereafter just called quarantine length) would help with management decision-making and planning, and could allow potential cost savings by having sufficient but not excessive resources available.

Currently, most programs extend quarantine periods past when the last fly is found, by calculating the amount of time required for a given number of generations (usually but not always three) to elapse under a thermal unit accumulation (“degree day”) physiological development model. Degree day based quarantine lengths have been codified in some legal regulations, including United States Federal code ^23^, California ^24^, and Florida. However, the procedure prescribed only defines when the end of a quarantine period has been reached after the fact. Additionally, the efficacy of pest surveillance efforts should factor into quarantine length, but that is beyond the scope of this paper.

For planning and resource allocation, policy makers and managers typically attempt to predict the quarantine lengths by using normal temperatures for forward projection. Although it frequently works fairly well, this approach is mathematically flawed and also provides no indication as to the variance or uncertainty of those predictions. Even a more rigorous treatment of degree day based values from historical temperature data can still produce highly variable results depending on relatively small changes in temperatures or details of the model formulation ^25^, in addition to neglecting important aspects of the biology.

Recently, another approach to determining effective quarantine durations against Medfly via Agent-Based Simulations (ABS) ^26^ was introduced. The MED-FOES system simulates a population of individual Medflies under inundative sterile insect technique (SIT) and other controls, explicitly modeling elimination as opposed to the degree day approach, which only determines the time for a specific number of generations to elapse to estimate quarantine duration. MED-FOES also allows for the sampling of parameter space (temperature dependent mortality for each stage, fecundity, etc.), producing a distribution of possible outcomes. While an ABS can be arbitrarily complex, MED-FOES is parameterized in such a way that it can model a ‘typical’ or hypothetical outbreak from only hourly temperature data, and is therefore similar to degree day methods in its input data requirements. It is also possible to vary the initial population to model a specific outbreak.

**Figure 1.**
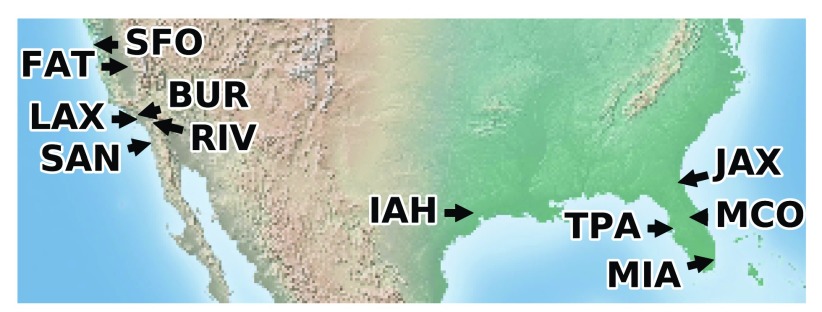
Locations of sites analyzed. Labels correspond to last three letters of the weather station callsigns in Table 1.

In this paper, predicted quarantine length (PQL) for 11 sites in the continental United States were analyzed ( Figure 1 and Table 1) based on both the standard thermal accumulation degree day method ^27^ as well as the MED-FOES ABS ^28^. Seasonal variation dominates quarantine duration, so we aggregated the PQL values for each day of the year (Jan. 1, Jan. 2, etc.) across a large number of years (65 for most locations) to produce normals. This approach enables comparison of the standard degree day method to the ABS, but more importantly provides insight into seasonal and spatial variations, prediction uncertainties, and model reliability.

**Table 1.** Weather station sites used (NOAA ISD).

Callsign	Station Name (as shown in data records)	State	Latitude	Longitude	Elevation	Start year
KSFO	SAN FRANCISCO INTERNATIONAL A	CA	+37.620	-122.365	2.4	1950
KFAT	FRESNO YOSEMITE INTERNATIONAL	CA	+36.780	-119.719	101.5	1950
KBUR	BURBANK-GLENDALE-PASA ARPT	CA	+34.201	-118.358	236.2	1973
KLAX	LOS ANGELES INTERNATIONAL AIR	CA	+33.938	-118.389	29.6	1950
KRIV	MARCH AIR RESERVE BASE	CA	+33.900	-117.250	468.2	1950
KSAN	SAN DIEGO INTERNATIONAL AIRPO	CA	+32.734	-117.183	4.6	1950
KJAX	JACKSONVILLE INTERNATIONAL A	FL	+30.495	-81.694	7.9	1950
KIAH	G BUSH INTERCONTINENTAL AP/HO	TX	+29.980	-95.360	29.0	1970
KMCO	ORLANDO INTERNATIONAL AIRPORT	FL	+28.434	-81.325	27.4	1973
KTPA	TAMPA INTERNATIONAL AIRPORT	FL	+27.962	-82.540	5.8	1950
KMIA	MIAMI INTERNATIONAL AIRPORT	FL	+25.791	-80.316	8.8	1950

## Methods

### Sites and temperature data

Hourly air temperature data for 11 sites was downloaded from NOAA’s publicly available Integrated Surface Database (ISD) dataset ^29, 30^.

The airport sites shown in Figure 1 were chosen for their biological relevance and availability of high quality hourly data over a long time frame. Models indicate that these sites are in regions suitable for Medfly ^31, 32^. Many of the sites experienced outbreaks in their vicinity the past and are of current concern. Additionally, they cover a range of conditions latitudinally as well as the California sites varying from coastal to more arid inland locations.

Sites are referred to here by the last three letters of the callsign shown in Table 1. For 8 sites (SFO, FAT, LAX, RIV, SAN, JAX, TPA, and MIA), temperature data starting on 1950-01-01 was used. The 3 other sites contained large ( *>*14 days) gaps or other problems in the early years of their data, so data starting on 1970-01-01 for IAH and 1973-01-01 for BUR and MCO was used. For all sites, temperature data from the start date through 2017-05-15 was used to generate PQLs for dates ranging from the start date to 2016-01-01.

Data was fetched and parsed using the Fetching and parsing ISH.ipynb ^*^ program. Records for the same station callsign were merged, since identification, format, and precise location of stations has changed over time. The data was then cleaned using the Cleaning temperatures.ipynb ^*^ by removing outliers, identifying large gaps ( *>* 3 hours), resampling to every hour on the hour using linear interpolation, and filling the large gaps using day-over-day linear interpolation (interpolating using values for the same hour of day from previous and following days). The resulting temperature datasets are available ^*^.

### Degree-day calculation

Degree-days were computed by the single-sine method ^27^, using a base development temperature of 12.39°C (54.3°F) and 345.56 degree-days Celsius (DDc; 622 DDf) per generation following the standard set by California Department of Food and Agriculture regulation 3406(b) ^24, 33^. Since hourly temperature data are available, we also calculated degree-days by simple summation for comparison ^25^. For each date, the number of days required for 3 generations of degree-day based life cycles was computed. These calculations are implemented in Temperature functions.ipynb ^*^.

### Agent-based simulations: MED-FOES

MED-FOES ^26, 28^ is an agent-based simulation explicitly modeling the eradication of a population of Medflies under inundative sterile male releases (sterile insect technique or SIT) and other interventions, such as increased trapping and foliar sprays. A MED-FOES simulation models a single non-spatial population, starting from a given population size and age distribution, tracking the number of individuals through time until the last fly (Agent) dies and the population is eliminated. In addition to hourly temperatures, simulation parameters include: the initial population, additional mortality induced by control efforts, the effectiveness of SIT, and a large number of biological parameters for which ranges are known from the literature including temperature-dependent development and mortality. The simulations were performed using the same hourly time series of temperature values used for degree-day calculations.

Due to the fact that many of the parameters are only known to within a range, 2500 individual MED-FOES simulations were run for each start date at each site, evenly sampling different regions of parameter-space via the Latin Hypercube Sampling ^34^ procedure. This set of simulations, encompassing a range of possible elimination outcomes, is referred to as a ‘run’. For example, each run include simulations with the initial number of adult females in the population ranging from 33 to 100, but the initial population age distribution was the same for all simulations. Initial population numbers were chosen as a “standard outbreak” based on seven real outbreaks modeled previously ^26^. LHS ranges for the probability of loss of reproduction due innundative SIT releases (0.5 to 1 chance per day) and additional human induced mortality from control efforts (0.05 to 0.15 per day) were chosen based on estimates of a typical California intervention ^26^. The full list of parameters used and their values is provided in Supplementary Table S1. The number of days from the start date required for 95% of the simulations in a run to be eliminated is taken as a conservative prediction of needed quarantine length and referred to as ABS PQL.

It is important to note that the 95% threshold for ABS PQL does not mean that there is a 95% chance a given outbreak will be eliminated. Instead, it refers to 95% of the LHC sampled points in parameter space reaching eradication by a given time. Despite the fact that we only know most of those parameters to within a range, it is almost certainly true that extreme values are less probable than mid-range values, and even more improbable that combinations of extreme values (for example: low mortalities and high fecundity) which lead to long eradication times will be as frequent as the uniform sampling the LHC procedure produces. Therefore, the 95% threshold used here is expected to be quite conservative.

Varying the start date for different simulations was achieved by simply starting at different points in the input temperature file; for this study a run was started every 7 days over the range of dates available for each site. Each set of runs for a single site over a range of starting dates is referred to as a ‘runset’. All runsets were conducted with the same input parameters aside from temperature. The 7 day interval ABS PQL values were upsampled to daily values using linear interpolation to allow day-of-year aggregations across years and comparisons with daily degree day based PQLs.

MED-FOES version 0.6.2 was run under Open Grid Scheduler/Grid Engine 2011.11 on a CentOS 6.6 HPC cluster. The MED-FOES code, configuration files, helper scripts, and raw results are available ^*^. Overall, we created 11 runsets (one for each site). Each runset contained runs starting every 7 days over the input temperature data range for that site, and each run contained 2500 individual simulations sampling different regions of biologically plausible parameter space. This sums to a total of approximately 86 *×*10 ^6^ simulations.

### Statistical analysis

The main results reported here are ‘normals’ in a meteorological sense of the term, but without the typical running mean smoothing which would complicate interpretation. For a variable of interest (eg. temperature or PQL), all values for the same calendar day irrespective of year (eg. 20-July) are aggregated, and summary statistics such as mean, minimum, maximum, and standard deviation are computed for each aggregation. Temperature functions.ipynb ^*^ contains the code used to perform normal calculations, and the code generating figures as well as all statistical analysis is Summary Figures.ipynb ^*^ (Jupyter Notebook ^35^, module and version information documented in the file).

The results reported here are the normals of PQL, computed using the full temperature time series as opposed to computing PQL from the normal of the temperature time series. While the latter is fairly common practice, it is not mathematically proper since, as with means, the normal of a function of *X* is not generally equal to the function applied to the normal of *X*. Additionally, by computing the normals of the predicted quarantine durations, we can investigate properties of the distribution of values as shown in Figure 3 and Figure 4 and the “supernorm" Supplementary Figure S1, Supplementary Figure S2, and Supplementary Figure S3.

## Results

**Figure 2.**
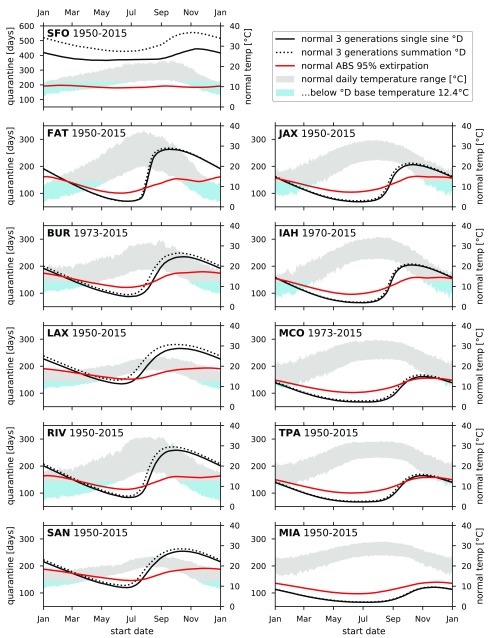
Summary of normal predicted quarantine length for each site and start date (last fly detection). Year range of input temperature data used is inclusive. All panels have identical limits except SFO quarantine.

**Figure 3.**
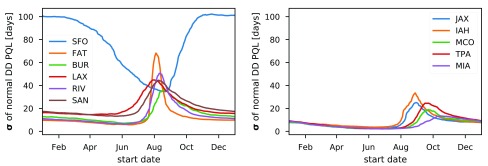
Variation in predicted quarantine length for each site and start date based on 3 generations of single-sine degree day accumulation.

**Figure 4.**
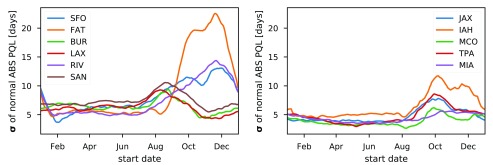
Variation in predicted quarantine length for each site and start date based on 95% of MED-FOES agent-based simulations showing elimination.

Figure 2 shows the mean of the normal PQL based on 3 generation degree day accumulation and MED-FOES 95% elimination along with the minimum and maximum of the normals for temperatures. Figure 3 and Figure 4 show the standard deviations ( *σ*) of the normals for the degree day and ABS based PQL.

There is significant variation in PQL across both time and location. The temporal variation in PQL is dominated by a yearly cycle, characterized by the normal values shown in Figure 2. Table 2 shows the percentage of variance in quarantine length predictions captured by the mean of the normal yearly cycle ( *R* ^2^) for each site. At all but one site, greater than 75% of the variance in both degree day and ABS based PQLs is accounted for by the mean normal, and the majority exceed 90%. SFO is an exception to this common trend, with the mean normal accounting for only 9.1% of the variation in degree day based PQL and 28.0% of the ABS based PQL. This is also reflected in supplementary figure S2 and supplementary figure S3.

**Table 2.** Percentage of PQL variance captured by the mean of the normal. DD PQL is the 3 generation single sine degree day based prediction, and ABS PQL is the MED-FOES agent-based simulation predictions.

Site	DD PQL *R* ^2^	ABS PQL *R* ^2^
SFO	9.12%	28.01%
FAT	93.93%	75.68%
BUR	90.71%	90.88%
LAX	80.17%	83.07%
RIV	92.23%	81.89%
SAN	80.99%	80.91%
JAX	96.45%	94.78%
IAH	95.10%	91.80%
MCO	94.62%	95.77%
TPA	91.91%	94.40%
MIA	88.42%	92.00%

### Seasonal dependence

Seasonal variation, evidenced by the general shape of the curves shown in Figure 2, is doubtless familiar to anyone engaged in Medfly pest management. Outbreaks starting in the late summer, autumn, or early winter will extend through relatively cold periods, when thermal dependent development will be slow and therefore extend the duration of quarantine required for 3 generations of degree days to accumulate (referred to as DD PQL hereafter). Similarly, outbreaks starting in the spring or early summer often lead to short quarantines due to the relatively high temperatures.

This familiar pattern is also seen in the ABS PQLs despite it being quite different in nature from simple degree day accumulation. However, the ABS predictions show a smaller seasonal swing. The ABS generally produces a smaller overall range of PQLs, with longer quarantines than DD PQL for spring and early summer outbreaks, and shorter quarantines for late summer through early winter in almost all cases.

A particular feature of interest, shown most dramatically at FAT in Figure 2, is that ABS PQL often flattens out or even dips for quarantines starting in the late autumn or early winter. This can be due to relatively rare and brief cold-snaps, normally lasting only a few hours, which increase mortality. Since DD PQL does not account for mortality, it misses the effect of cold-snaps entirely. This effect is most clearly seen at more northern and inland sites where cold-snaps are more likely: particularly FAT and RIV, but also BUR, LAX, JAX, and IAH.

### Geographic dependence

PQL generally shows a positive correlation with latitude, and sites are ordered by latitude in the figures and tables here. As seen in Figure 2, higher latitude sites tend to have longer PQLs as well as larger seasonal swings for both degree day and ABS based predictions.

**Figure 5.**
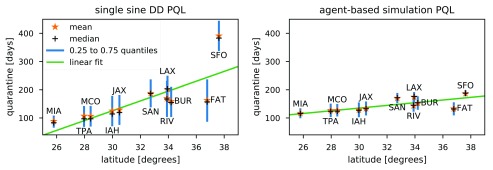
Predicted quarantine length dependence on latitude. For each site, the mean, median, and inter-quartile range are shown, similar to a boxplot. An ordinary least-squares linear fit to the median values is shown by the green lines. The left panel is for single sine degree day predictions, and MED-FOES ABS based predictions are in the right panel.

Figure 5 shows the relationship between PQL and latitude. An ordinary least squares fit to the median PQL at each site shows a significant slope for both DD PQL ( *F* =14.08, *p*=0.005) and ABS PQL ( *F* =10.55, *p*=0.010), but the degree day based predictions are more sensitive to latitude than the ABS (coefficients of 17.39 and 4.78 respectively). Additionally, the ABS predictions are more stable for SFO, and to a lesser extent FAT, where the degree day model for Medfly produced PQLs that appear either unrealistically long (SFO) or are subject to rapid and extreme seasonal variation in the mid year (FAT).

In addition to the variation associated with latitude, large differences in PQLs computed for the same start date can exist between even relatively nearby sites. For example, the differences in both degree day and ABS PQLs for the three sites in the Los Angeles region (LAX, BUR, RIV) (shown in the supplementary figure S4) display a strong seasonal component with a spike in July and/or August. The difference in DD PQL between LAX and BUR is normally about a month (overall median=35 days; overall 25% & 75% quantiles are 28 & 45 days), but the median difference of the normal exceeds 75 days in August with some PQL differences up to 142 days. Differences in ABS PQLs are more seasonally stable, with the LAX minus BUR difference not exceeding 42 days for any start date in the 43 years analyzed here.

### Variance and uncertainty

Figure 3 and Figure 4 report the standard deviation ( *σ*) of the normal for DD PQL and the MED-FOES ABS PQL respectively. These indicate the year to year variability of the PQL for outbreaks starting at a given time of the year and can be used to gauge the uncertainty of predictions based on past PQLs relative to the actual quarantine length which will be required. Similar information is represented by the inter-quartile ranges shown in Figure 5 and supplementary figure S2 and supplementary figure S3. The distributions of PQL values for a site and day-of-year (aggregating across years) are generally not highly skewed, making *σ* a relatively easy to interpret measure of uncertainty.

Excluding SFO, the mean normal is a good predictor of DD PQL with *σ* values below 20 days except for the late summer and early autumn, where variance increases due to quarantines extending through the cold season. FAT and, to a lesser extent, RIV show this increase more dramatically, presumably due to their more arid/inland climates where both daily and seasonal temperature ranges are larger (also see Figure 2). The standard deviation generally decreases with decreasing latitude, together with reduced means. The standard deviation in DD PQL for SFO shows an inversion of the seasonal trend other sites exhibit. This is due to the colder temperatures leading to extremely long DD PQLs, frequently extending across two winter seasons.

The standard deviations of the ABS PQL normals shown in Figure 4 are generally about 1/2 as large as for DD PQL. This indicates that the ABS PQL not only shows less dramatic seasonal swings, but is also produces more consistent predictions across years. Values again generally decrease with latitude, but less consistently than DD PQL *σ* of normals. Also, unlike with the DD PQL, the results for SFO appear consistent with other sites.

A notable feature is that BUR, LAX, and SAN all show an increase in the year to year variation in ABS PQLs starting in July and extending through November, while that increase for all other sites starts in July or August but extends to January or February. Additionally, results for FAT show a sharp increase in uncertainty starting in September, fitting with the more arid/inland climate. RIV shows a significant but more gradual increase.

### Historical quarantines

Thirty-four Medfly quarantines in CA dating from 1975 to early 2017 were analyzed ( supplementary table S2). The start of all but two of these quarantines was in the latter half of the year (July through December), when DD PQLs are typically relatively long, with 68% (23 */*34) occurring in September through October, when DD PQLs are longest. August, the month where uncertainty in DD PQL often spikes (see Figure 3), accounts for 30% (7 */*34) of historic quarantines.

For each historic quarantine start date, the DD PQL and ABS PQL for the closest of the 11 sites analyzed above (see Figure 1 and Table 1) to the actual outbreak location was determined (see supplementary table S2). For this set of hypothetical quarantines, the ABS produced significantly shorter quarantines (mean=169.7 days, *σ*=21.8 days) than simple 3 generation degree day accumulation (mean=234.2 days, *σ*=79.2 days) ( *df* =33, *t*=6.01, *p*<10 ^*−*5^). Additionally, the variance in the difference between quarantine lengths using a specific date and the mean of the normal PQL for that day of year was smaller for the ABS ( *σ*=8.2 days) than with degree day ( *σ*=25.9 days) ( *df* =33, *F* =9.92, *p*<10 ^*−*8^).

## Discussion

The principal contributions of this work can be broken down into three categories:

1) Comparison of PQLs as determined by the degree day and ABS methods.
2) Variation in average PQLs across time of year and space; and
3) Variation in PQLs within a time of year and location.

Consideration of all three of these by program managers, planners and other decision makers is likely to improve management of Medfly incursions by informing resource allocation ahead of outbreaks, reducing quarantine costs in some cases, and reducing risk from premature quarantine suspension in others. The results presented cover most of the latitudinal range of Medfly suitability within the United States, as well as many sites of probable introduction, and will hopefully find use as a general guide. Eradication models are extremely difficult to test for accuracy given the impracticality of experimental introductions and the sparse and idiosyncratic nature of historic outbreaks. However, analyzing the timing and locations of historic outbreaks suggests that quarantine lengths would generally be more consistent and shorter on average in California if estimated by ABS compared with degree day.

Requiring a fixed number of generations (typically 3) of degree days to pass is a “tried and true" method, but not explicitly an extirpation model. It may overestimate required quarantine length through cold weather ^26^ and may underestimate length when growth conditions are very favorable, which somewhat paradoxically leads to shorter degree day based quarantine periods after the last fly detection since generation times are shorter. However, the simplicity of the degree day calculation is a point in its favor, together with its record of generally avoiding subsequent detections after eradication measures and quarantine establishment ^21^.

ABS results may be used to inform and modulate responses and treatments such as delimination trapping, fruit sampling, and eradication measures which are under the some discretion of managers. In situations where DD PQL greatly exceed those from the ABS, it is likely that degree day is missing important effects, such as cold snaps, which may justify shortening quarantine periods. On the other hand, in cases where the ABS predicts longer times to elimination the degree day indicated quarantine may be unusually short, so treatments and SIT releases should be conducted more aggressively than normal to ensure eradication is achieved within the perscribed degree day based quarantine.

A few specific results arising from overall comparisons of different locations are worth highlighting. In general, DD PQLs for Medfly generated from San Francisco International Airport temperature data are almost certainly too long for the entire year. The ABS PQLs are flatter and seem more realistic at around 200 days for San Francisco compared with the 400–550 days of DD PQLs. For several other California locations (typified by Fresno and Riverside) DD PQLs are in close alignment with those from the ABS for the first half of the year but go significantly longer in the cooler months. For three of the four Florida locations analyzed, DD PQLs are significantly shorter than the ABS results (Miami, Tampa, and Orlando). The extent of the difference in those Florida locations is smaller in the later months of the year, but the generality of this pattern suggests that the margin of safety for quarantines as calculated by degree day in those locations may be smaller than expected.

There is significant variation in PQL depending on the location of the outbreak, with the extremes in our study sites represented by Miami and San Francisco. These geographic results could be compared to previous efforts to model climatic suitability of different parts of the US. One of the early studies on the subject focused on Medfly found higher climatic suitability in Florida locations (Fort Pierce and Orlando) compared with California sites ^36^. Within California, however, those authors found a higher number of suitable months in coastal areas such as Oceanside compared with Riverside and Fresno, roughly paralleling our findings (compare Los Angeles or San Diego with Fresno or Riverside). A more recent analysis of climatic suitability likewise concludes that coastal S. California is the most favorable area of the state for Medfly, but favorability drops inland in the south due to desert conditions. Suitability in central and northern California is limited by cold temperatures and freezes ^31^.

An important aspect of ABS PQLs is variation within particular times of years and locations. Rare events like cold snaps can increase mortality in the ABS, and thereby lead to shorter PQLs than expected based on historical averages, or DD PQLs. The specificity of the ABS is helpful for determining when quarantines might be safely suspended due to such a rare event not be captured by the degree day model. For cold temperatures especially there can be a significant difference in PQLs: The degree day model includes only development, which is halted at low temperatures, extending quarantine lengths. The ABS, however, also includes mortality for generating PQLs, which means that low temperatures can significantly reduce estimates. Historically in California, quarantines have most frequently occurred at times of year when degree day based quarantines are drawn out by cold weather and the MED-FOES ABS model predicts significantly shorter durations. Furthermore, 30% of those historic quarantines happened in August where there is a great deal of uncertainty in forward predictions of degree day quarantine durations based on normal values. If we assume those historic CA quarantines are a guide, the ABS model would very likely produced more predictable and shorter quarantine durations for future outbreaks.

Combination of the two methods analyzed here could leverage the best aspects of both methods for determining optimal quarantine length. The initial quarantine length estimate could be quickly produced via degree-day calculation or the ABS based on the distribution of PQL values generated using historical temperatures. This would generate not just a single “typical” value as the current method of projecting using historical average/normal temperatures does, but a range of outcomes. The median “most likely” value may be used for official estimates, while the variance and extremes would provide managers and affected parties additional information vital for planning.

Once the three generation period has started after the last fly find, weekly ABS simulations could indicate the likelihood that the pest has been successfully eliminated. If 95% of simulations show elimination, the decision to end quarantine early could be made, or in the case where the ABS has not reached the 95% threshold at the end of the DD PQL additional measures could be considered to reduce the risk of re-detection.

## Data and software availability

All data, non-standard programs, and scripts used area available in the GitHub repository: https://github.com/travc/paper-Predicted-MF-Quarantine-Length-Data-and-Code, archived at https://doi.org/10.5281/zenodo.1006698. Files are documented in the repository’s README, and the analysis scripts ( .ipynb files) are viewable online at GitHub. Efforts were made to make the code understandable. It is our intent that someone with a reasonable level of programming knowledge will be able to not only replicate our analysis, but also use portions of the provided code as a basis for their own analysis.

## Footnotes

* See the Data and software availability section

## Supplementary material

**Figure S1: Daily normal of hourly temperatures.** Hourly temperature data aggregated by day of year.

**Figure S2: Degree day based PQL supernorm.** Single sine degree day based predicted quarantine lengths aggregated by day of year.

**Figure S3: MED-FOES PQL based supernorm.** MED-FOES based predicted quarantine lengths aggregated by day of year.

**Figure S4: Difference in PQL at nearby sites (LA basin example).** The difference in PQL values for the same start date (including year) between sites in the LA basin. For each date over the range of available data (1973 through 2015 for LAX-BUR and RIV-BUR, 1950 through 2015 for LAX-RIV), PQL values for each site are computed and the differences taken. The resulting differences are then aggregated by day-of-year. Lines show the median while the shaded region is the 25% to 75% quantile range for each day-of-year aggregation of differences.

**Table S1: MED-FOES parameters.** Parameter values or Latin-Hypercube Sampling ranges used for running MED-FOES simulations.

**Table S2: Historical quarantines.** Historical quarantines in California.
