# Supplementary material for: Evaluation of predicted Medfly ( Ceratitis capitata) quarantine length in the United States utilizing degree-day and agent-based models
Source: F1000Res. 2018 Mar 6;6:1863. Originally published 2017 Oct 20. [Version 2] doi: 10.12688/f1000research.12817.2 (PMC5773928; doi:10.12688/f1000research.12817.2)
Supplement: Supplementary file 1 [file f1000research-6-15340-s0000.tgz › 41cf5c67-d0f5-4e61-9c26-c9e78eedf48b.pdf]

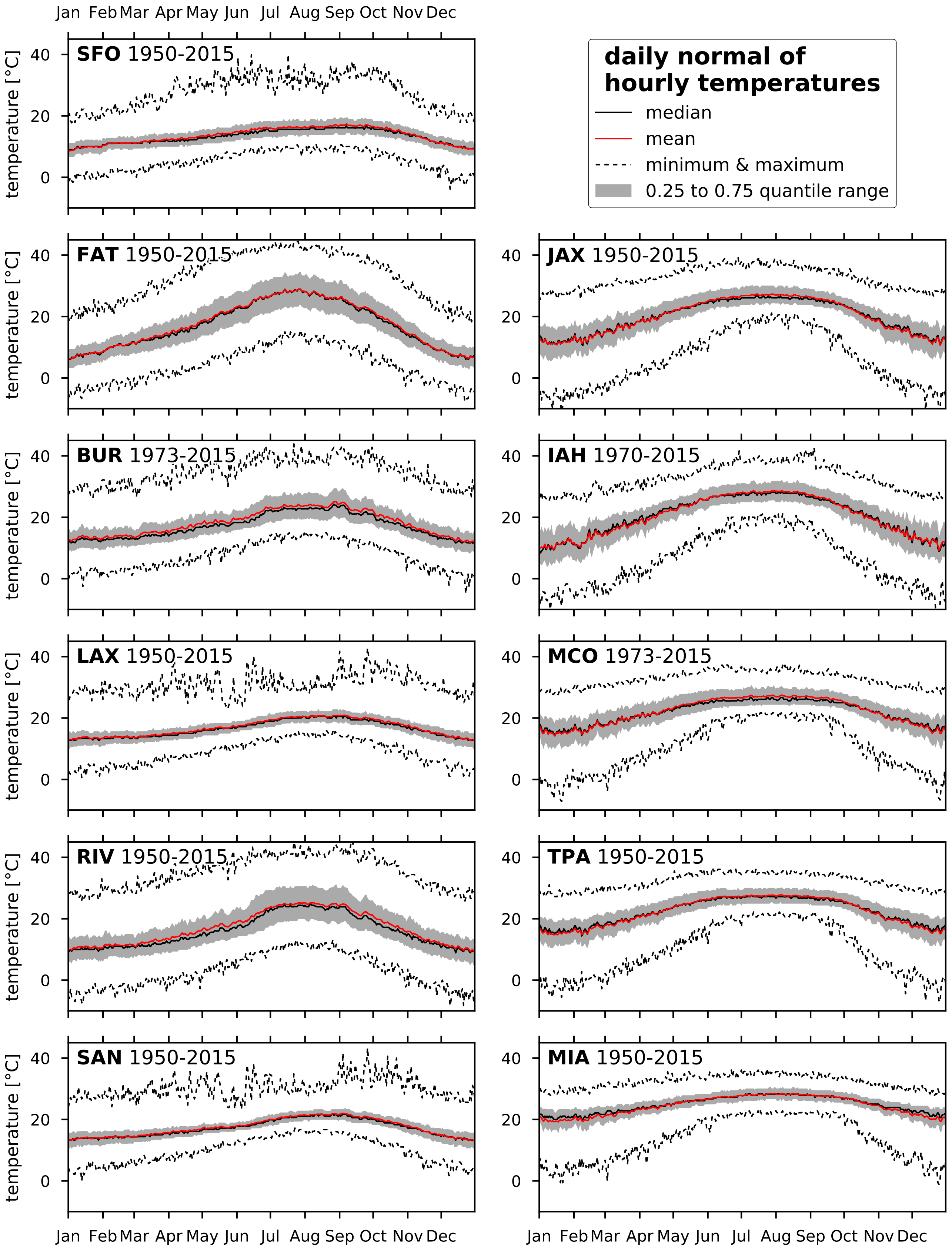
daily normal of hourly temperatures
median
mean
minimum & maximum
0.25 to 0.75 quantile range
SFO 1950-2015
FAT 1950-2015
JAX 1950-2015
BUR 1973-2015
IAH 1970-2015
LAX 1950-2015
MCO 1973-2015
RIV 1950-2015
TPA 1950-2015
SAN 1950-2015
MIA 1950-2015
temperature [°C]
Jan Feb Mar Apr May Jun Jul Aug Sep Oct Nov Dec
