# Supplementary figures and images for: Evaluation of predicted Medfly ( Ceratitis capitata) quarantine length in the United States utilizing degree-day and agent-based models
Source: F1000Res. 2018 Mar 6;6:1863. Originally published 2017 Oct 20. [Version 2] doi: 10.12688/f1000research.12817.2 (PMC5773928; doi:10.12688/f1000research.12817.2)

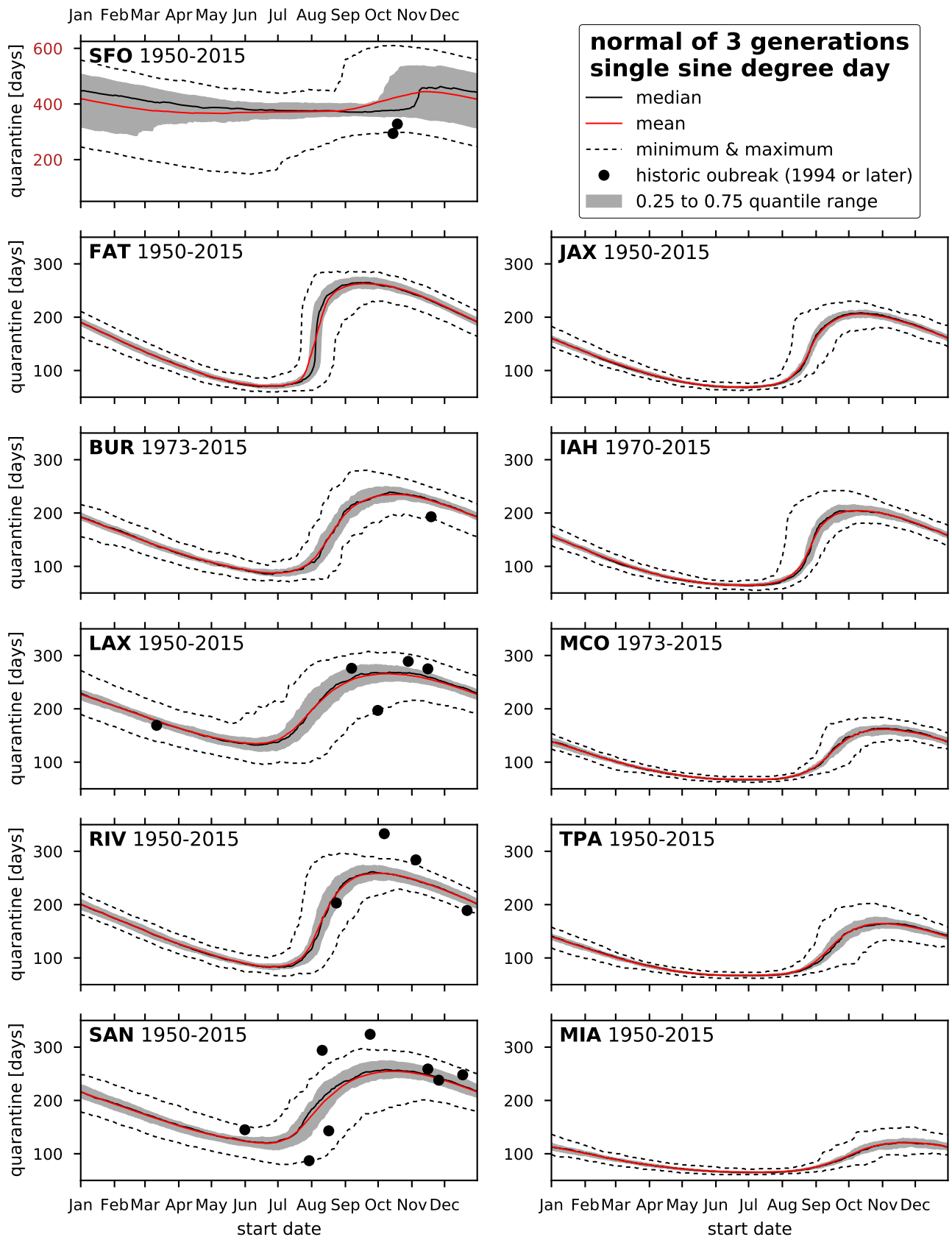

Supplement: Supplementary file 2 [file f1000research-6-15340-s0001.tgz › 5e38aa1d-69cf-452a-a517-3137bfa8ad5a.pdf]

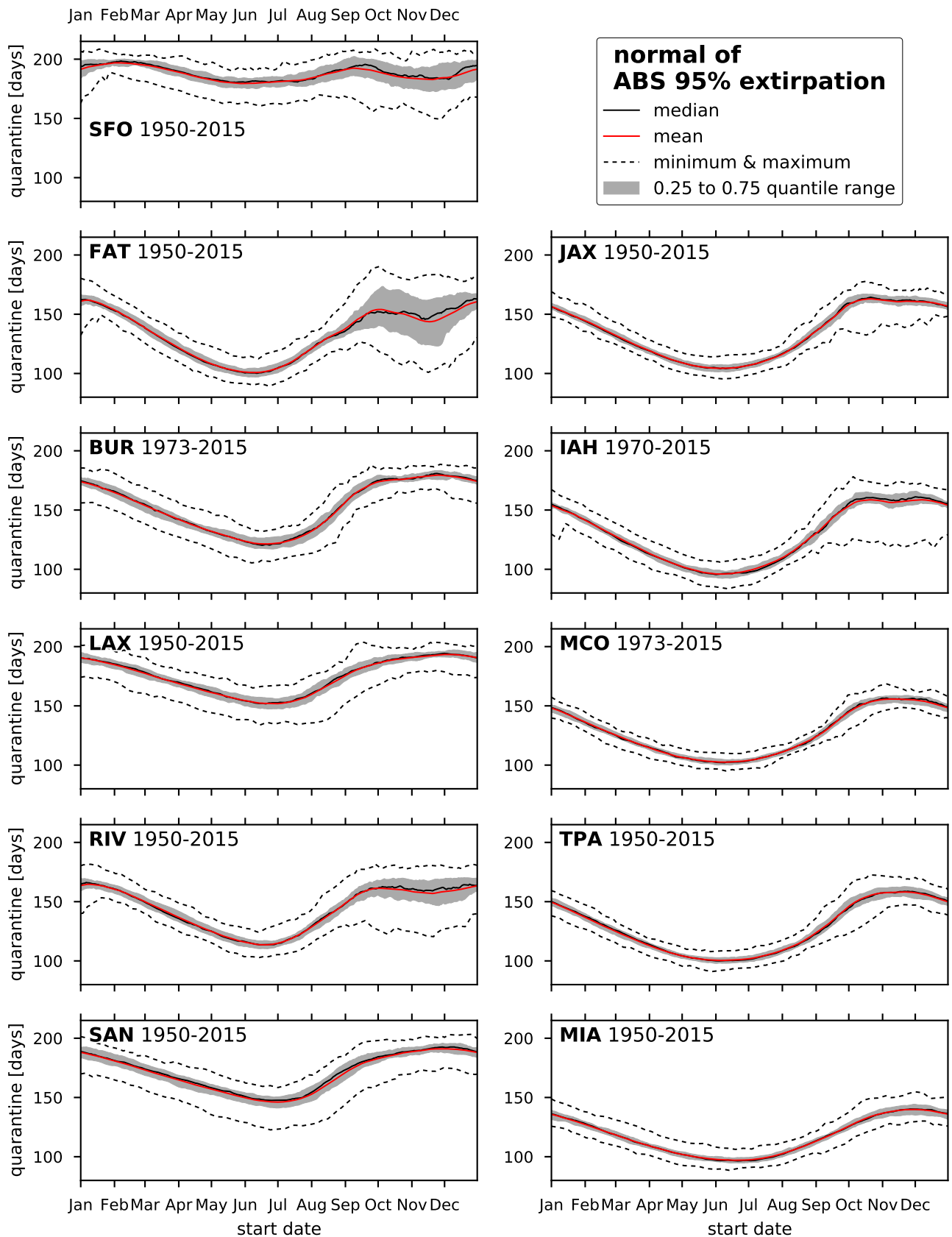

Supplement: Supplementary file 3 [file f1000research-6-15340-s0002.tgz › 94b3d6de-5576-435a-a908-8ad76c6ed601.pdf]

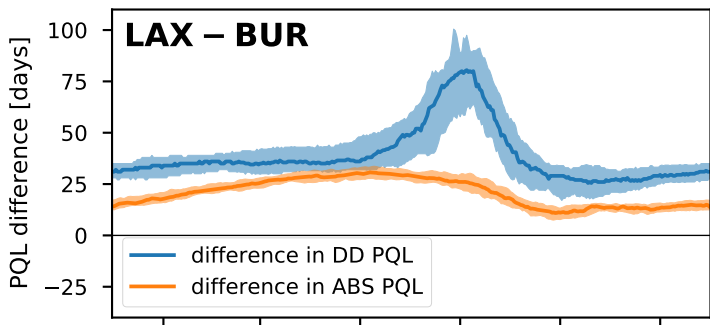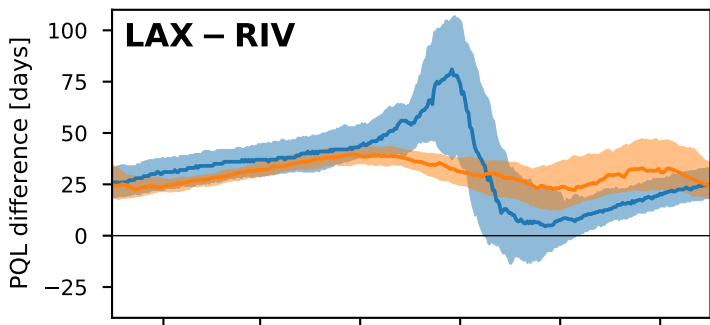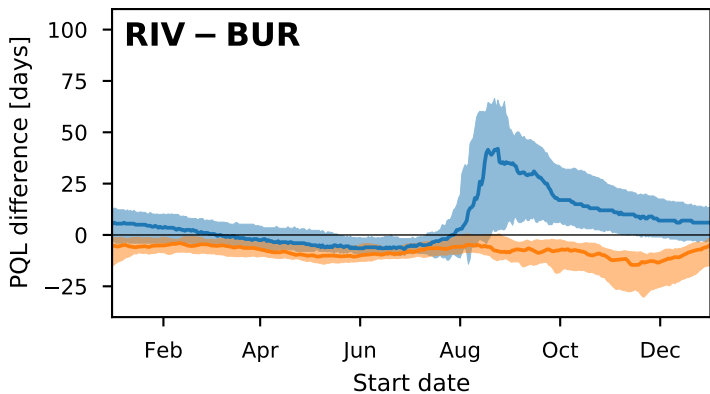

Supplement: Supplementary file 4 [file f1000research-6-15340-s0003.tgz › 0d10b491-af7e-400a-9c0f-ef2c51af9168.pdf]
