# Supplementary material for: Evaluation of predicted Medfly ( Ceratitis capitata) quarantine length in the United States utilizing degree-day and agent-based models
Source: F1000Res. 2018 Mar 6;6:1863. Originally published 2017 Oct 20. [Version 2] doi: 10.12688/f1000research.12817.2 (PMC5773928; doi:10.12688/f1000research.12817.2)
Supplement: Peer review history

# Peer review: Referee response for version 2

**DOI:** 10.5256/f1000research.15340.r31530

**Published:** 2018-03-16

**Authors:** John M. Kean

**Affiliations:** 1 Forage Systems, AgResearch, Christchurch, New Zealand

I thank the authors for addressing my feedback on the first version of the paper. Most of my reservations were adequately addressed. A few grammatical or typological errors remain (e.g. “metain-vasion”, “data” treated as a singular) but these are very minor concerns. There is, however, one point that I think warrants a follow-up comment from the authors.

 Fruit fly responses typically involve heightened trapping for monitoring eradication. The 3-generation guideline operates from the time that the last insect is detected. Similarly, the agent-based simulation (ABS) implicitly assumes that no further fruit flies are detected - otherwise this would influence decision-making around the appropriate quarantine period. However, there might be some simulations in which the ABS population becomes large, and these would most likely be those that persist for the longest and have the greatest chance of generating further detections. This might introduce a slight bias in the results, such that the ABS potentially overestimates the quarantine time required since the last detection. This does not at all alter the conclusions from the study, but it does highlight the importance of surveillance trapping in informing eradication decision making.

I have read this submission. I believe that I have an appropriate level of expertise to confirm that it is of an acceptable scientific standard.

# Peer review: Referee response for version 1

**DOI:** 10.5256/f1000research.13887.r29759

**Published:** 2018-01-24

**Authors:** John M. Kean

**Affiliations:** 1 Forage Systems, AgResearch, Christchurch, New Zealand

I thoroughly support the intent of this paper: to bring additional accuracy and rigour to biosecurity decision making. Specifically, the question of “when is eradication achieved” is an interesting and important one. It is not easy to address, however, but this paper makes an important contribution.

 Biosecurity aware economies have recognised the need for science-based reform of international practices around quarantines ^1^. Current practice has largely withstood the test of time, but is based on simplistic assumptions. As this paper points out, better decisions might be made with the better biological data and population dynamic tools now available.

 This paper describes a well-designed comparison between an existing “rule of thumb” approach and a more biologically savvy modelling approach. The strengths and weaknesses of each method are alluded to and the reasons for misalignment explored. The authors suggest a balanced approach to utilise the strengths and minimise the weaknesses of each. On the whole, I support the study and its findings, with two caveats.

 First, some additional details are needed to fully understand what the agent-based simulations (ABS) are doing. Critical information is lacking on the temperature relationships and simulated management in the model. While this information would be available from the computer code provided, I am loathe to search through 423 MB of source code to find it. Without these details I cannot make a confident assessment of the study.

 Second, the study highlights the importance of low temperatures in fruit fly phenology, but glosses over some of the biologically relevant complications. More detailed comments on the paper sections are given below.

 Introduction

 The examples given for eradications are all historical, including the cited review paper ^2^, which is not very optimistic about eradication as an effective tool for managing invasions. The science of eradication has advanced considerably in recent years and better understanding of invasive ecology, improved surveillance and control tools, and important advances in understanding and managing social expectations around eradication programmes mean that biosecurity agencies can now conduct such operations with much greater certainty, efficacy and efficiency. I suggest replacing the cited review paper ^2^ with Liebhold *et al*. (2016) ^3^ which gives a more up-to-date review of eradication science.

 I note that the quarantine guideline of three generations is not universally used. For example, Australia uses one generation plus thirty days.

 I am intrigued by the statement that predicting generation times into the future using normal temperatures is “mathematically flawed” and would like further clarification about what the authors mean.

 Methods

 It would be very useful to specify the developmental parameters used by the ABS, especially if they differ from those used for the day-degree approach. I note that the day degree parameters used in California differ considerably from other published values, which find a base development temperature of 8 to 10°C ^4^.

 No details are given about the assumed starting populations and age structures for ABS runs. This seems a critical detail. Also, what were the assumed management conditions? Without management the simulated populations would presumably increase (on average) and the fact that eradication was achieved in the simulations suggests that some sort of management must have been in place. Details of this are critical for understanding what the simulation results mean and how applicable they may be to different cases.

 In addition, I feel an important aspect of quarantine has been completely ignored – that of surveillance efficacy. The relevant international guidelines (ISPM 6, 9 and 26) specify that surveillance and monitoring are a key part of any programme aiming to prove freedom from a pest. For fruit flies, surveillance trapping is used to monitor eradications, with the quarantine period applying from the time of the last confirmed detection. Therefore surveillance effort is a critical factor in determining an appropriate quarantine length. If surveillance were perfect then no quarantine period would be needed because we would know that nothing is there. Conversely, if surveillance is poor then a large population might conceivably persist undetected for a very long time, necessitating a very long quarantine period indeed. I assume that the ABS simulated the surveillance practices used in California, but some details are needed. I believe that fruit fly surveillance practices differ between different parts of California – could this explain part of the differences in results between sites (Figure 2)?

 Results

 The ABS results were tallied as the simulated times after which 95% of simulated populations were eradicated. Depending on the situation, regulator might prefer greater (e.g. 99%) or lesser certainty. It would be useful to see an example of a population survival curve to understand how quarantine duration affects the risk of non-eradication. I suspect that such information would be very useful for biosecurity authorities in their decision-making.

 Figure 2 has a spelling mistake – “extripation”.

 Conclusions

 A key point in the paper is the hypothesis that cold snaps may help to knock out fruit fly populations more quickly than expected by the simple day-degree method. The implication is that the ABS method may enable significant cost savings in such situations by allowing a substantially shorter quarantine period. This is a valuable insight, but should be tempered by the fact that fruit flies may have special tricks to enable them to survive cold snaps. The torpor and cold survival thresholds for Queensland fruit fly, for example, are conditioned by previous exposure to low temperatures ^5^ ^6^, an effect that I suspect is not captured in the ABS. Therefore, I would urge some caution in applying the ABS recommendations in such situations.

 Over all, Medfly development and survival at relatively low temperatures seems to be a key factor in setting quarantine periods that start in late summer/autumn, and in understanding the different predictions from the day-degree and ABS methods. However linear day degree models, as used in both approaches, are known to have questionable validity near the predicted developmental threshold. Also (as noted by the authors) small changes in the threshold temperature parameter might cause relatively large changes in the predicted quarantine times. This is not a particular issue for this study, but is a common problem in the simulation of insect phenology, especially in temperate climates. Given the economic importance of fruit flies, better data and models for behavioural and physiological thresholds could be very useful for making better biosecurity decisions.

I have read this submission. I believe that I have an appropriate level of expertise to confirm that it is of an acceptable scientific standard, however I have significant reservations, as outlined above.

## References

[1] OrmsbyMD: Evaluation of Import and Export Parameters for Fruit Fly Export Restriction Zones. *New Zealand Ministry or Primary Industries: Technical Document*.2016;

[2] MyersJHSimberloffDKurisAMCareyJR: Eradication revisited: dealing with exotic species. *Trends Ecol Evol*.2000;15(8) :316-320 1088469510.1016/s0169-5347(00)01914-5

[3] LiebholdAMBerecLBrockerhoffEGEpanchin-NiellRSHastingsAHermsDAKeanJMMcCulloughDGSucklingDMTobinPCYamanakaT: Eradication of Invading Insect Populations: From Concepts to Applications. *Annu Rev Entomol*.2016;61: 10.1146/annurev-ento-010715-023809 335-52 10.1146/annurev-ento-010715-023809 26667377

[4] KeanJM: Meta-analysis, validation and application of fruit fly development times. *New Zealand Plant Protection*.2015;68:44-53

[5] MeatsA: Seasonal trends in acclimatization to cold in the Queensland fruit fly (Dacus tryoni, Diptera) and their prediction by means of a physiological model fed with climatological data. *Oecologia*.1976;26(1) : 10.1007/BF00345654 73-87 10.1007/BF00345654 28309105

[6] MeatsA: Critical periods for developmental acclimation to cold in the Queensland fruit fly, Dacus tryoni. *Journal of Insect Physiology*.1983;29(12) : 10.1016/0022-1910(83)90057-4 943-946 10.1016/0022-1910(83)90057-4

# Peer review: Referee response for version 1

**DOI:** 10.5256/f1000research.13887.r29994

**Published:** 2018-01-18

**Authors:** Daniel M. Borchert

**Affiliations:** 1 Plant Protection and Quarantine, Center for Plant Health Science and Technology, Plant Epidemiology and Risk Analysis Laboratory, United States Department of Agriculture (USDA)-Animal and Plant Health Inspection Service (APHIS) , Raleigh, NC, USA

This research was well done and provides valuable analysis and insight into an ongoing challenge that is faced in monitoring fruit fly quarantines. 

 One point of note is the base developmental threshold reported for the Medfly DD model of 53.3 F should be 54.3 F. I am not sure if this was a typo in the manuscript or if it was carried through the calculations. This would likely impact the length of the PQL for the degree day models in both the lower and higher latitude areas, but I do not think that the results would be dramatically different.  I recommend that this be checked and revised if necessary.

 In my opinion, the ABS models do provide an improvement to the estimations of Predicted Quarantine Length and can be a beneficial tool in the regulatory area.

I have read this submission. I believe that I have an appropriate level of expertise to confirm that it is of an acceptable scientific standard.

# Response

**Published:** 2018-01-18

**Authors:** Travis Collier

**Affiliations:** US Department of Agriculture, Agricultural Research Service, USA

Good catch on base temp...  It is fortunately just a typo.  The code used to do all the calculations, which is posted on Github, has the correct 54.3'C value.

We will correct that and address Teresa Vera's comments in a revision shortly.

Thank you

Travis

# Response

**Published:** 2018-01-18

**Authors:** Travis Collier

**Affiliations:** US Department of Agriculture, Agricultural Research Service, USA

I meant 54.3'F, not 'C in the above comment.  Sorry

 For the record, the degree-day model used is:

 Base temp = 54.3'F (12.3889'C)

 DD per generation = 622 DDf (345.5556 DDc)

 from "Ceratitis capitata- Review of Degree Day Models" (2011): USDA APHIS Medfly Action Plan

 also in CDFA regs sec 3406: https://www.cdfa.ca.gov/plant/medfly/docs/regs/3406-TXT-medfly.pdf

 The original source appears to be:

 Tassan, R. L., K. S. Hagen, A. Cheng, T. K. Palmer, G. Feliciano and T. L. Bough. 1982.

 Mediterranean fruit fly life cycle estimations for the California eradication program.

 CEC/IOBC Symposium Athens November 1982. pp. 564-570

 Of possible note:

 An alternative model used by Chile and I suspect other places, which might be more applicable to coastal central CA is from Grout and Stoltz (2007):

 Base temp = 9.9'C

 DD per generation = 338 DDc

# Peer review: Referee response for version 1

**DOI:** 10.5256/f1000research.13887.r27623

**Published:** 2017-11-21

**Authors:** Teresa Vera

**Affiliations:** 1 Instituto Universitario UHM-CEAM, Valencia, Spain

The article explains the significance of using two different simulation models to calculate quarantine length after repeated pest invasions, particularly from *Ceratitis capitate*. Quarantine length is a key issue: non- detection doesn’t mean the pest invasion has been eliminated. Therefore, authors have selected eleven sites in the USA to compare quarantine lengths estimated with the usual simulation model –called degree-day model- and a newer one –called Agent-based simulation (ABS) in order to compare both. The first approach calculates quarantine lengths taking into account the time needed to pass 3 pest generations and a thermal approach. The second approach calculates the lengths considering population and elimination.

 I think it’s a very interesting paper showing how important is the approach selected in order to determine quarantine lengths. The comparison of both approaches gives some useful information about which approach could suit better in function of season, latitude and longitude etc. When both methods are combined, managers can select the best aspects of each one to optimize quarantine lengths. As authors remark at the end of Discussion, using this combination, vital information for planning is provided to managers and affected parts.

 Only a few recommendations:

 In my opinion, selection of sites is not well explained. Authors mention the sites “were chosen for their biological relevance and availability of high quality data”, the last is easy to understand; however the first is not so obvious. I’d recommend a brief explanation of why airports have a biological relevance.

 Regarding Statistical analysis, I recommended a longer explanation: it seems like the only parameter compared is temperature, what about ABS inputs (as initial population, mortality and so on)?

 I like very much the detailed information provided in the Results section.

 I’d recommend some changes in the Discussion section since discussion of the results is put together with conclusions. It could be interesting to add a Conclusion section after Discussion, and then the article structure would be clearer.

 Finally, reference sources tend to be quite old; I’d recommend authors to update them, at least some of them.

I have read this submission. I believe that I have an appropriate level of expertise to confirm that it is of an acceptable scientific standard.

## References

[1] CollierTManoukisN: Evaluation of predicted Medfly (Ceratitis capitata) quarantine length in the United States utilizing degree-day and agent-based models. *F1000Research*.2017;6: 10.12688/f1000research.12817.1 10.12688/f1000research.12817.1 29399322PMC5773928

# Response

**Published:** 2017-12-15

**Authors:** Travis Collier

**Affiliations:** US Department of Agriculture, Agricultural Research Service, USA

Thank you for the review and useful recommendations.  Apologies for the long delay in responding.  We are planning to address your review in detail along with an additional review and posting a revised paper, but that second review is taking a long time to come in.

 I don't want your contribution to go too long without being acknowledged though.

 Thank you
